# Role of GDNF, GFRα1 and GFAP in a *Bifidobacterium*-Intervention Induced Mouse Model of Intestinal Neuronal Dysplasia

**DOI:** 10.3389/fped.2021.795678

**Published:** 2022-01-14

**Authors:** Wei Liu, Tingting Zhou, Jinqiu Tian, Xiaofang Yu, Chuantao Ren, Zengcai Cao, Peimin Hou, Qiangye Zhang, Aiwu Li

**Affiliations:** ^1^Department of Pediatric Surgery, Qilu Hospital, Cheeloo College of Medicine, Shandong University, Jinan, China; ^2^Department of Pediatric Surgery, Dezhou People's Hospital, Dezhou, China

**Keywords:** intestinal neuronal dysplasia (IND), gut microbiota, *Bifidobacterium*, homozygous mutant mice of Tlx2, GDNF

## Abstract

**Objective:**

To investigate the effects of glial cell-derived neurotrophic factor (GDNF), GDNF family receptor alpha 1 (GFRα1), and glial fibrillary acidic protein (GFAP) on colonic motility in a mouse model of intestinal neuronal dysplasia by intervention with *Bifidobacterium* and to explore the influence of *Bifidobacterium* on enteric glial cells (EGCs).

**Methods:**

Western blotting and qRT-PCR were employed to detect the expression of GFRα1 and GFAP in colonic tissues of mice with or without Tlx2 mutations, and ELISA was used to detect the expression of GDNF in serum. IHC was used to detect the appearance of the ganglion cells. Subsequently, Tlx2 homozygous mutant (Tlx2^−/−^) mice were treated with *Bifidobacterium*. Colonic motility was measured before and after intervention by measuring the glass bead expelling time. The variations in abdominal circumference and GDNF, GFRα1, and GFAP expression were measured. In addition, 16SrRNA gene sequencing was performed to detect the abundance of the intestinal microbiota.

**Results:**

The mRNA and protein expression of GFRα1 and GFAP was decreased in the colonic tissues of Tlx2^−/−^ mice and GDNF expression was decreased in serum compared with Tlx2^+/−^ and WT mice. After confirming the colonization of *Bifidobacterium* by 16S rRNA gene sequencing, the expelling time and abdominal distension were ameliorated, and the expression of GFAP, GDNF, and GFRα1 was increased.

**Conclusions:**

The expression of GDNF, GFRα1, and GFAP is associated with colonic motility. The altered expression of EGC-related factors suggested that *Bifidobacterium* may be involved in the EGC activation process. The amelioration of IND symptoms after intervention with *Bifidobacterium* prompted the elicitation of adjuvant therapy.

## Introduction

Intestinal neuronal dysplasia (IND) is a disorder of the enteric nervous system (ENS) associated with intestinal dysmotility (1). Similar to Hirschsprung's disease (HSCR), patients with IND show severe prolonged defecation time, constipation, abdominal distension, and even intestinal obstruction. However, the pathological features of IND are distinct, and include: hyperplasia of the submucosal nerve plexus, immaturity of ganglia, and hypertrophy of the nerve trunks in clinical cases (2, 3). Since the first description by Meier-Ruge in 1971 (4), the definition of IND is still a subject of controversy. Although the histopathological diagnostic criteria for IND are continuously updated, its etiology and pathogenesis have not been elucidated. Glial cell line-derived neurotrophic factor (GDNF), secreted by enteric neuroglial cells (EGCs), are regarded as one of the most important neurotrophic pathways in the formation of the ENS (5). GDNF binds to GDNF family receptor alpha 1 (GFRα1), causing phosphorylation of the tyrosine kinase receptor RE-arranged during transfection (RET). Signals through the activation of downstream signaling pathways direct the development of the ENS, including neuroblast migration and axonal outgrowth (6, 7).

Gut microbiome can affect the function and number of enteric neurons (8–10), and the early gut microbiota plays an important role in the development of the ENS after birth (11, 12). Several studies have shown that abnormalities in the myenteric plexus and a decrease of intestinal motility can be observed in early postnatal germ-free mice (11). While there are hundreds of species of bacteria in the human intestine, *bifidobacteria* is a key microbial player in the infant gut microbiota and has a beneficial effect on gut microbiota composition, leading to reduced necrotizing enterocolitis (NEC) incidence (13, 14). Furthermore, *bifidobacterium* can affect the expression of inflammatory factors in EGCs to inhibit intestinal inflammation and upregulate the expression of enteric glial cell–derived nerve growth factor (NGF) and neurotrophin 3 (NT-3) (15). These findings prompted us to determine whether colonic dysmotility of IND can be improved by the probiotic components of intestinal flora through activation with EGCs.

To verify this hypothesis, Tlx2 homozygous mutant (Tlx2^−/−^) mice were chosen as the model. T-Cell Leukemia Homeobox Protein 2 (Tlx2), a member of orphan homeobox-containing transcription factor family, is also known as HOX11L1, Ncx and Enx (16). Tlx2 has been proved to be crucial to the development of ENS. Tlx2^−/−^ mice show colonic dysmotility with hyperplasia of intestinal ganglion and immature enteric neurons after birth, which has been confirmed to be consistent with IND (17, 18). Glial fibrillary acidic protein (GFAP), a type III intermediate filament (IF) protein that is highly expressed in mature and activated EGCs (19–21), was chosen as a marker of EGCs. This study aimed to explore the effects of GDNF, GFRα1, and GFAP on colonic motility and the influence of *Bifidobacterium animalis* on EGCs, and to further clarify the potential pathogenesis of IND.

## Materials and Methods

### Animal and Sample Preparation

The study was approved by the Ethics Committee of Qilu Hospital of Shandong University (IACUC Issue No. Dull-2020-013). The mice were treated according to the animal use guidelines of the Animal Care and Use Committee (ACUC) of Qilu Hospital, Shandong University. Genechem (Shanghai, China) used CRISPR/Cas9 gene-targeting technology to knock out 173 bp of the second exon nucleotide sequence of the C57BL/6 mouse Tlx2 gene, resulting in Tlx2^+/−^ mice. Tlx2^+/−^ mice were interbred to obtain Tlx2^−/−^ mice. The mice were reared under specific pathogen-free conditions. At the age of 3 weeks, different genotypes were screened out through genotype identification (WT Tlx2^+/−^ and Tlx2^−/−^). Colonic motilities and abdominal circumference were measured 1 d before or 1 d after the intervention. Feces were collected 1 d after the intervention and before colonic motility measurement. Blood samples and segments of the distal colon were harvested after anesthesia, and were stored at −80°C.

### Genotyping

The genotype of mice was identified by Southern blotting. Then the mice were divided into three groups (group WT Tlx2^+/−^ and Tlx2^−/−^, nine mice for each group) according to their genotypes for experiments before probiotic intervention. Genomic DNA was isolated from mouse tails using a Mouse Tail DNA Extraction kit (CWBio, Beijing, China). The primer sequences for Tlx2 are shown in Table 1. Subsequently, the genomic DNA was amplified by PCR and separated by 1.2% agarose gel electrophoresis. Images were acquired using the BIO-RAD gel image acquisition system (Bio-Rad, Hercules, CA, USA).

**Table 1:** Detailed primer information.

**Primer**	**Species**	**F sequences (5^**′**^-3^**′**^)**	**R sequences (5^**′**^-3^**′**^)**
Tlx2	mus	TTGATGAGGCTTCTGTGGTT	AAGAGCGACGAGTTGTGC
GFAP	mus	TAACGACTATCGCCGCCAAC	CATTTGCCGCTCTAGGGACT
GFRα1	mus	CTATCGTCCCTGTGTGCTCC	CCAATCAGTCCCGAGTAGGG
GDNF	mus	GTCACCAGATAAACAAGCGGC	CTCTGCGACCTTTCCCTCTG
GAPDH	mus	TGTCTCCTGCGACTTCAACA	GGTGGTCCAGGGTTTCTTACT

### Measure of Mouse Colonic Motility

Colonic motility was measured 1 d before or 1 d after the intervention. A small glass bead with a diameter of 2.5 mm was inserted slowly into the colon of the mice to a distance of 2 cm with a smooth glass rod (2.5 mm diameter). After checking that the glass bead was completely pushed in, the mouse was placed on a clean surgical drape, and the expulsion was recorded.

### *Bifidobacterium animalis* Intervention

*Bifidobacterium animalis* (AS1.1852, biobw) was cultured in *Bifidobacterium* nutrient liquid medium (Haibo, Qingdao, China) in an anaerobic incubator at 37°C, and Spectroscopy Photometric Microplate Reader (ThermoFisher, MA, US) was used to monitor the density of the bacterial colony (OD 600). The bacterial colony density was adjusted to 1 × 10^9^ CFU/ml before intervention. Tlx2^−/−^ mice with a suitable weight at the age of 3–4 weeks were divided into three groups (eight mice for each group). *Bifidobacterium animalis* group (group BB) was given live *Bifidobacterium* liquid according to the weight of each mouse by retention-enema at 0.2 ml/20 g (0.2 × 10^9^ CFU/20 g) daily for 3 weeks. The solution should be prepared before each intervention everyday and used in time after dilution to maintain the probiotic activity. The *Bifidobacterium* nutrient medium group (group NM) was administered the same dose of nutrient medium, and the normal saline/blank control group (group NS) was administered the same dose of normal saline.

### Western Blotting

Western blotting was used to analyze the relative expression levels of GFRα1 and GFAP proteins in the colonic tissue. The Minute™ Total Rapid Protein Ext Kit (Invent, MN, USA) was used to isolate total protein from the full-thickness colonic tissue. Equal amounts of protein were separated on a 10% SDS-PAGE gel and then transferred to a PVDF membrane. The PVDF membrane was incubated with primary antibodies at 4°C overnight after 5% BSA blocking. The membrane was then washed with 1 × TBST and incubated with secondary antibody for 1 h at room temperature. ECL kit (Millipore, MA, USA) was used for chemiluminescence, and the gray values were calculated. Table 2 shows the details of the antibodies.

**Table 2:** Detailed antibody information.

**Antibodies**	**Company**	**Species**	**Dilution**	**Cat. No**
GFAP	Affinity	Rabbit	WB 1:1000 IHC 1:500	AF6166
GFRα1	Abcam	Rabbit	1:800	Ab8026
GAPDH	Proteintech	Rabbit	1:1000	10494-1-AP
IgG (H + L)	Affinity	Goat	WB 1:5000 IHC 1:200	S0001

### Quantitative Real-Time PCR

The qPCR assay was employed to investigate the relative expression of GDNF, GFRα1, and GFAP in colon tissue at the mRNA level. Total mRNA was isolated using TRIzol reagent (Ambion, USA). After concentration measurement, mRNA was reverse transcribed to cDNA using an RT-PCR kit (Toyota, Japan). The qPCR reactions were performed with SYBR Green PCR Master Mix (Toyota, Japan) on a Roche 480 real-time fluorescent PCR instrument. Primers were selected from the Beacon Designer software (Premier Biosoft, Palo Alto, California, USA). The detailed information is shown in Table 1. The 2^−ΔΔCt^ values for each group were calculated for analysis. Colon sample of each mouse was repeated three times, and a melting curve was used to confirm specificity.

### Immunohistochemistry Staining

Immunohistochemistry was used to detect the appearance of ganglion and glial cells. The colon tissue was fixed in 4% paraformaldehyde for 24 h and then embedded in 5-μm paraffin. After dewaxing and antigen retrieval, the slices were blocked with goat serum and incubated with the primary antibodies (1:500) overnight at 4°C, then incubated with secondary antibodies (1:200) for 1 h at 37°C. All antibody incubation and washing steps were performed in PBS at pH 7.4. DAB (ZSbio, Beijing, China) staining was monitored under a microscope. Finally, the sections were counterstained with hematoxylin and coverslipped. The integrated optical density (IOD) of positive staining within five random fields from paraffin sections of each mouse was measured at 20 × magnification using Image-Pro Plus 6.0 image analysis software (Media Cybernetics, Bethesda, MD, USA). The average value was used for statistics.

### ELISA

An ELISA kit (Boster, Wuhan, China) was used to detect the level of GDNF in serum according to the manufacturer's instructions. Serum was added to a 96-well plate (100 μl per well) for detection. The OD values were measured at 450 nm after the reaction, and the concentrations were calculated.

### 16S rRNA Gene Sequencing

Total bacterial DNA was extracted from fecal samples using the E.Z.N.A. Stool DNA Kit (Omega, Inc., USA). The V3-V4 region of the bacterial 16S rRNA gene was amplified using the universal primers 338F (5-ACTCCTACGGGAGGCAGCAG-3) and 806R (5-GGACTACHVGGGTWTCTAAT-3). PCR products were purified with AMPure XT magnetic beads (Beckman Coulter Genomics, Danvers, MA, USA), and quantified using Qubit (Invitrogen, California, USA). The amplicon library was prepared for sequencing, and its size and quantity were assessed using an Agilent 2100 Bioanalyzer (Agilent, CA, USA) and the Library Quantification Kit for Illumina (Kapa Biosciences, Woburn, MA, USA). The amplicon library was paired-end sequenced using the Illumina MiSeq platform at a commercial company (LC-Bio Technology Co., Ltd, Hang Zhou, China).

### Statistics and Analysis

All statistical analyses were performed using GraphPad Prism®8 software, and the results are presented as the mean ± SD. *T*-test was used for comparisons between two independent groups, and one-way analysis of variance (ANOVA) and Tukey's test were used for comparisons between multiple groups; *P* < 0.05 was considered statistically significant.

## Results

### Tlx2^−/−^ Mice Showed Decreases in the Expression of GDNF, GFRα1 and GFAP

**Figure 1:**
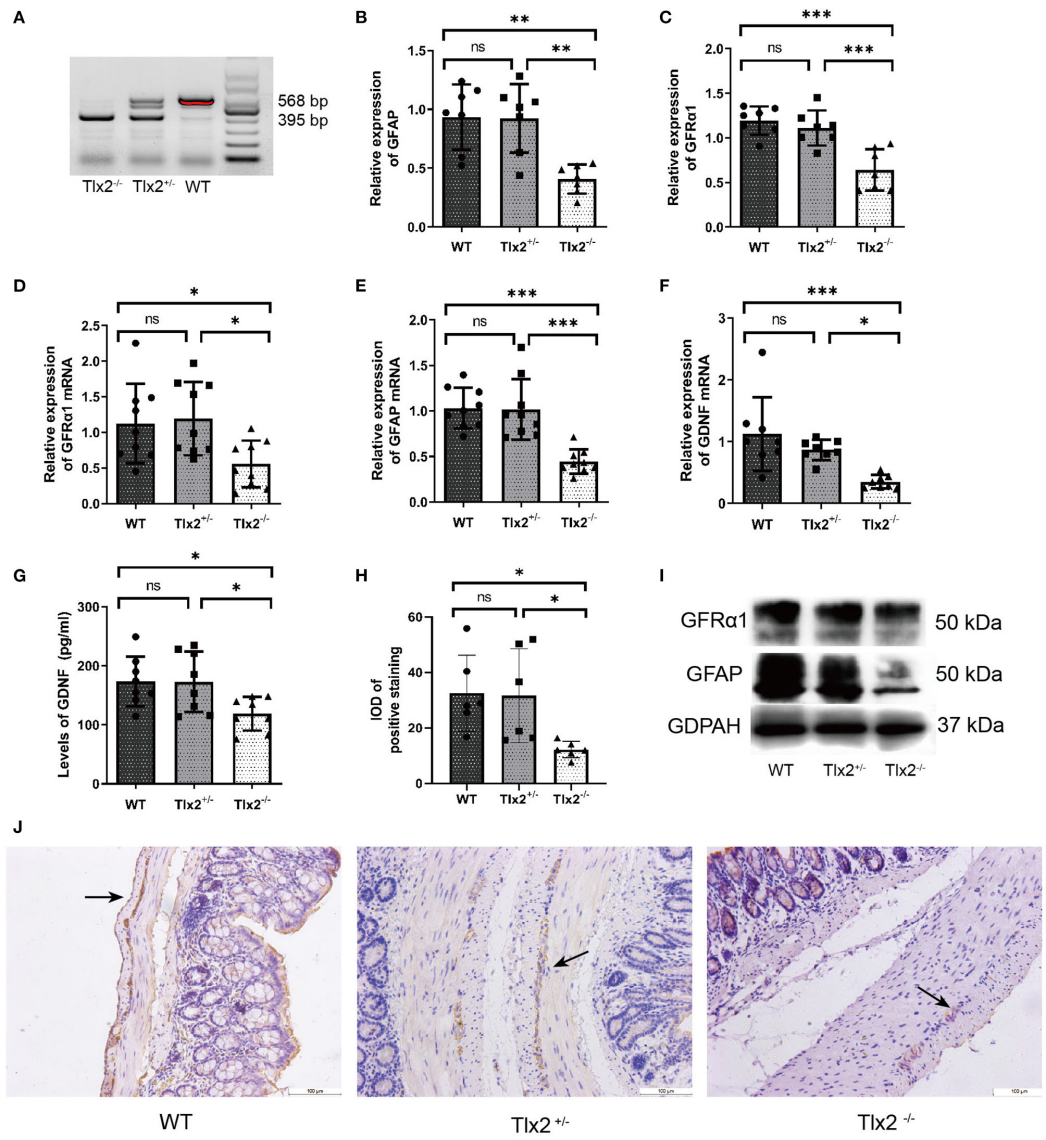
Example genotyping and decreased expressions of GDNF, GFRα1 and GFAP in Tlx2^−/−^ mice. **(A)** Genotyping; the wild-type allele is 568 bp, and the mutant allele is 395 bp. **(B,C)** Western blot indicated that the protein expressions of GFAP and GFRα1 in colonic tissues of Tlx2^−/−^ mice were decreased (*n* = 7 for each group). **(D–F)** The mRNA expressions of GDNF, GFRα and GFAP were decreased in colon tissues of Tlx2^−/−^ mice (*n* = 9 for each group). **(G)** The expression level of GDNF in serum of Tlx2^−/−^ mice was lower than that in Tlx2^+/−^ mice and WT mice (*n* = 8 for each group). **(H)** IOD of positive staining indicated that GFAP was significantly downregulated in the colonic tissues of Tlx2^−/−^ mice (*n* = 6 for each group). **(I)** Representative western blot analysis and detail bands of other mice were attached in Supplementary Figure 1. **(J)** GFAP was mainly expressed in EGCs of the colonic myenteric plexus (ns *P* > 0.05; *P < 0.05; **P < 0.01; ***P < 0.001; GAPDH, glyceraldehyde-3-phosphate; Scale bar: 100 μm).

The allele of the wild-type was 568 bp, whereas the mutant was 395 bp. T1x2 ^−/−^ mice obtained by interbreeding were genotyped by Southern blot analysis (Figure 1A). To confirm whether the expressions of GDNF, GFRα1, and GFAP were different among WT, Tlx2^+/−^, and Tlx2^−/−^ mice, the colonic samples and sera of these mice were used for analysis. The protein and mRNA expression of GFRα1 and GFAP in colonic tissues of Tlx2^−/−^ mice were lower than those in Tlx2^+/−^ and WT mice (*P* < 0.05) (Figures 1B–E,I). Compared to Tlx2^+/−^ mice and WT mice, the protein expression of GDNF in serum and its mRNA expression in colonic samples of Tlx2^−/−^ mice was also decreased (*P* < 0.05) (Figures 1F,G). Meanwhile, IHC staining showed that GFAP was mainly expressed in the EGCs of the colonic myenteric plexus (Figure 1J). The IOD of positive staining indicated that GFAP was significantly downregulated in colonic tissues of Tlx2^−/−^ mice compared to other tissues (Figure 1H).

### The Expelling Time of Tlx2^−/−^ Mice Was Shortened and the Abdominal Circumference Was Smaller After *Bifidobacterium* Intervention

**Figure 2:**
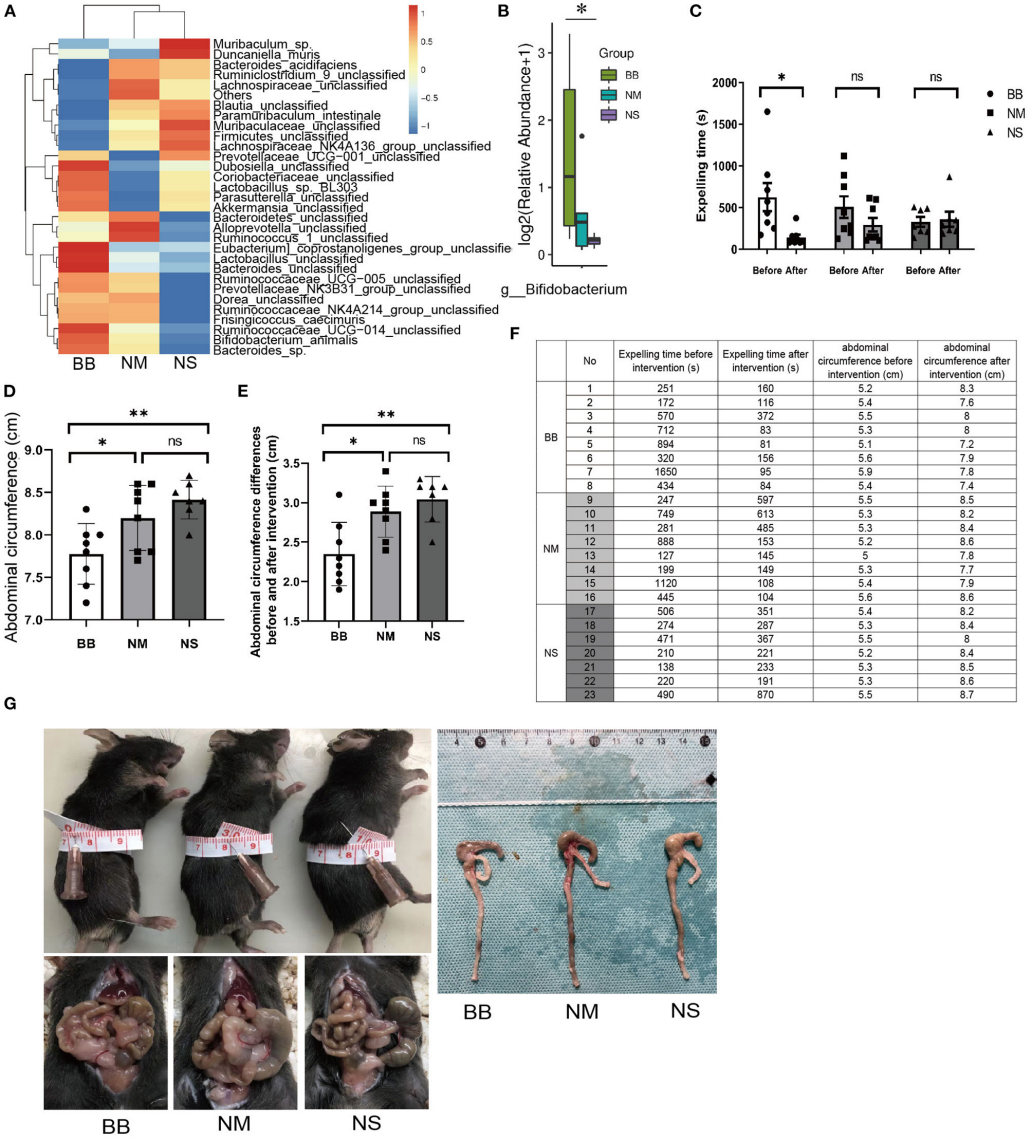
Measurement of the colonic motility and abdominal circumference of mice in groups BB, NM and NS. **(A)** Hierarchical clustering heatmap of the top 30 bacterial species of each group. **(B)** The relative abundance of *Bifidobacterium* among groups BB, NM and NS. **(C,F)** T After *Bifidobacterium* intervention, the expelling time in BB group was shortened compared to groups NM and NS. **(D,F,G)** The abdominal circumference after *Bifidobacterium* intervention among the three groups. **(E,F)** Differences in abdominal circumference before and after *Bifidobacterium* intervention in group BB was smaller than the other groups. (ns *P* > 0.05; *P < 0.05; **P < 0.01).

To confirm that the *Bifidobacterium* intervention was effective, 16S rRNA gene sequencing was performed. The relative abundances of intestinal flora were presented in a hierarchical clustering heatmap and *Bifidobacterium* in group BB was much higher than that in groups NM and NS (Figures 2A,B). The time of bead expulsion in different groups of Tlx2^−/−^ mice before and after enema was recorded (Figure 2F). The expelling time of group BB after enema was shorter than that before the intervention (*P* < 0.05). However, nutrient medium and normal saline did not affect colonic motility (Figure 2C). The abdominal circumference of mice in group BB after intervention was significantly smaller than that of the other two groups (*P* < 0.05) (Figures 2D,F) and the differences in abdominal circumference before and after *Bifidobacterium* intervention in the BB group were smaller than those in the other groups (*p* < 0.05; Figure 2E). The abdominal distension in the BB group was relieved (Figure 2G). The gross anatomy of the intestines revealed that the fecal retention of mice in group BB was mild, and the dilatation of the cecum, distal ileum, and proximal colon was relieved compared with other groups (Figure 2G).

### The Expression of GDNF, GFRα1 and GFAP Was Increased in Tlx2^−/−^ Mice After Intervention

**Figure 3:**
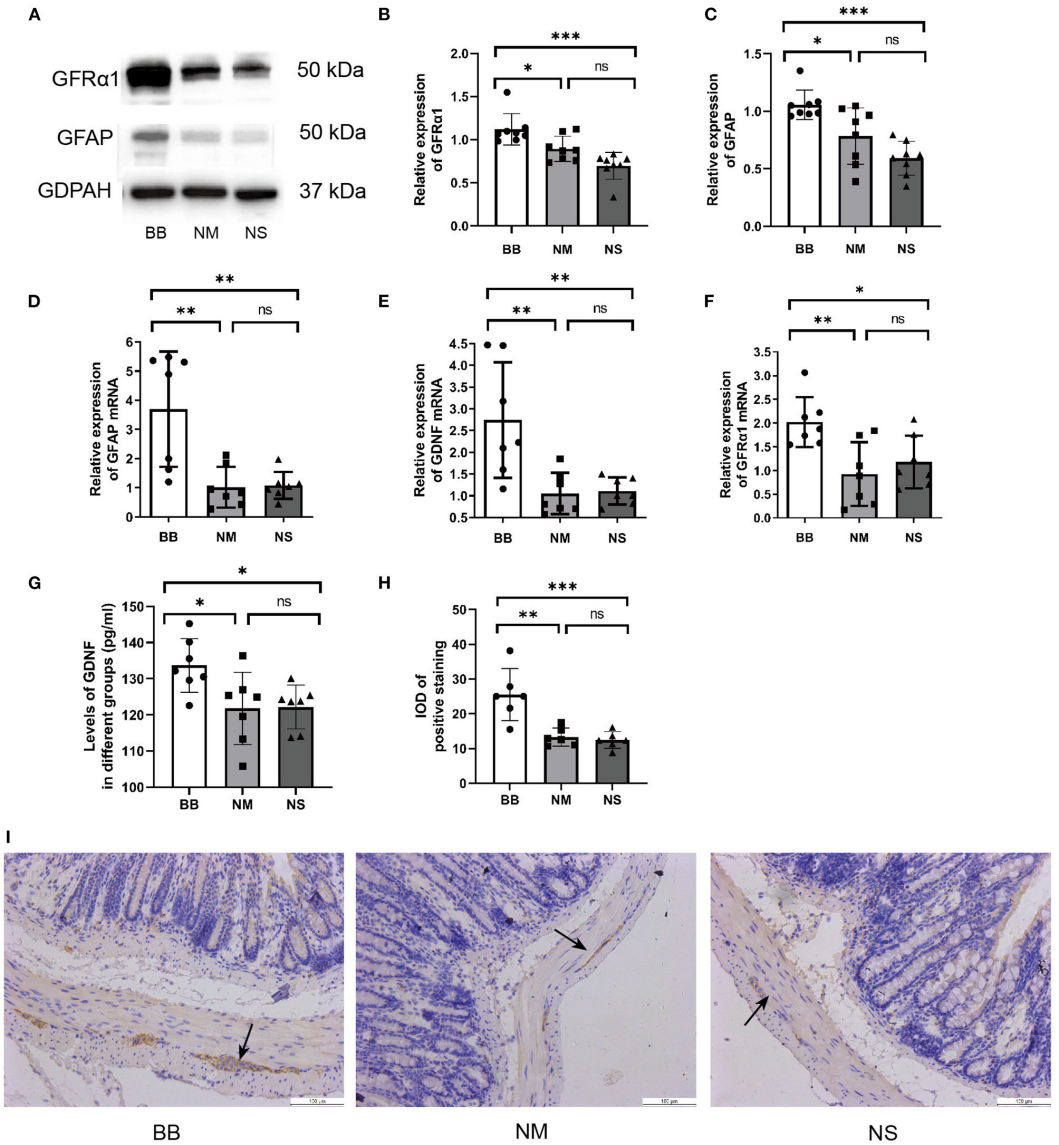
Expression of GDNF, GFRα1 and GFAP was increased in group BB. **(A–C)** The protein expression of GFAP and GFRα in the colonic tissues of group BB was higher than those in the other two groups (*n* = 8 for each group), detail bands of other mice can be found at Supplementary Figure 1. **(D–F)** The mRNA expressions of GDNF, GFRα and GFAP in colon tissues of group BB were increased (*n* = 7 for each group). **(G)** The serum levels of GDNF in group BB increased compared to those in groups NM and NS after intervention (*n* = 7 for each group). **(H)** The IOD of positive staining in group BB was increased compared to that of the NM and NS groups (*n* = 6 for each group). **(I)** Representative results of IHC staining of GFAP in different groups under 20 × microscope (ns *P* > 0.05; *P < 0.05; **P < 0.01; ***P < 0.001; GAPDH, glyceraldehyde-3-phosphate; Scale bar: 100 μm).

The protein expression of GFAP and GFRα1 was significantly increased in the BB group (Figures 3A–C), which was consistent with the qRT-PCR results (Figures 3D,F). ELISA was used to detect the expression level of GDNF in the serum, confirming the upregulated expression of GDNF in the BB group (Figure 3G). Similarly, the qRT-PCR assay revealed that mRNA expression of GDNF increased in the colonic tissues of mice in the BB group (Figure 3E), and there was no statistically significant difference between the other two groups. The location and expression of GFAP are shown by IHC (Figures 3H,I). As can be seen, the stain-positive cells of GFAP presented tan granules. The IOD of positive staining in group BB was higher than that in groups NM and NS, which was consistent with the results of WB and PCR.

## Discussion

The pathogenesis of IND is unresolved; however, several mechanisms have been proposed, including secondary infections, inflammation, and developmental failures (22–24). It has been confirmed that the seriously impaired colonic motility of IND is related to abnormal innervation between ENS and intestinal smooth muscle cells (25, 26). Tlx2^−/−^ mice, a verified model of IND, usually show hyperplasia of the intestinal ganglion and persistence of immature enteric neurons after birth (18, 27). In our previous studies, Tlx2^−/−^ mice showed obvious abdominal distension and impaired colonic motility, and 26% (33/127) of the mice died 8 weeks after birth (25), which may be related to histopathological changes in the colonic tissues of Tlx2^−/−^ mice.

The main origin of the ENS is neural crest cells (28). EGCs and enteric neurons, vital components of the ENS, show interactive influences during the maturation and function of the ENS (28), and GDNF and GFRα1 play significant roles in this process (6, 7, 29). In this study, the expression of GDNF, GFRα1, and GFAP in Tlx2^−/−^ mice was lower than that in Tlx2^+/−^ and WT mice, confirming that the three may participate in the pathogenesis of IND. Alternatively, we observed higher expression of GDNF, GFRα1, and GFAP in mice after intervention with *Bifidobacterium*. In addition, the expelling times in group BB were significantly shortened. All of these results indicate possible relationship between GDNF, GFRα1, and GFAP and the relief of colonic motility dysfunction, which might explain the colonic motility dysfunction resulting from the immaturity of ganglion cells.

The gut microbiota affects the activation of the immune system by modulating antigen-specific adaptive immune responses, resulting in physiological functions (8, 30). Bacteria metabolites regulate the intestinal microenvironment by modulating the release of inflammatory factors (31). *Bifidobacterium*, the main probiotic component of the gut microbiota in humans, can reduce inflammatory responses by inhibiting the NF-κB and external cell signaling pathways (32). It has been confirmed that EGCs can be regulated by *Bifidobacterium* through inhibition of inflammation and up-regulation of nerve growth factors *in vitro* (15). In our study, the *Bifidobacterium* intervention in IND model mice caused the colonization of *Bifidobacterium* and increased expression of GDNF, GFRα1, and GFAP in colonic tissues. The variations in the expression of GDNF, GFRα1, and GFAP suggested that *Bifidobacterium* may participate in EGC activation and nutritional factor upregulation, but the specific mechanism of interaction between *Bifidobacterium* and EGC should be further researched.

IND and HSCR show similar clinical features, including constipation, abdominal distension, and even intestinal obstruction. In this study, after intervention by *Bifidobacterium*, the expelling time of Tlx2^−/−^ mice was shortened and abdominal distension was reduced. The recovery of the impaired colonic motility confirmed the positive influence of *Bifidobacterium* on colonic motility, which is consistent with our clinical experience. These results are also consistent with recent findings that fecal microbiota transplantation can improve intestinal function (33, 34).

As shown above, the expression of GDNF, GFRα1, and GFAP decreased in Tlx2^−/−^ mice and increased after *Bifidobacterium* intervention, while the IND-related symptoms were relieved after *Bifidobacterium* intervention. We can conclude that the expression of GDNF, GFRα1, and GFAP is associated with colonic motility dysfunction and the pathogenesis of IND. The altered expression of EGC-related genes suggested that *Bifidobacterium* may be involved in the activation process of EGCs. The amelioration of IND symptoms after intervention with *Bifidobacterium* prompted the elicitation of adjuvant therapy.

## Data Availability Statement

The datasets presented in this article are not readily available because the dataset also forms part of an ongoing study. Requests to access the datasets should be directed to 591043945@qq.com.

## Ethics Statement

The animal study was reviewed and approved by the Ethics Committee of Qilu Hospital of Shandong University.

## Author Contributions

WL and TZ: design of the work, acquisition, analysis, and interpretation of data for the work. JT, XY, CR, ZC, and PH: feeding of laboratory animal and collection of specimens. QZ and AL: drafting the work or revising it critically for important intellectual content. All authors contributed to the article and approved the submitted version.

## Funding

This work was funded by the National Natural Science Foundation of China (Projects Nos. 81873846 and 82071682).

## Conflict of Interest

The authors declare that the research was conducted in the absence of any commercial or financial relationships that could be construed as a potential conflict of interest.

## Publisher's Note

All claims expressed in this article are solely those of the authors and do not necessarily represent those of their affiliated organizations, or those of the publisher, the editors and the reviewers. Any product that may be evaluated in this article, or claim that may be made by its manufacturer, is not guaranteed or endorsed by the publisher.

## Supplementary Material

Supplementary Figure 1 **(A)** Western blot indicated that the protein expressions of GFAP and GFRα1 in colonic tissues of Tlx2^−/−^ mice were decreased. **(B)** The protein expression of GFAP and GFRα in the colonic tissues of group BB was higher than those in the other two groups.
