# Supplementary figures and images for: Role of GDNF, GFRα1 and GFAP in a Bifidobacterium-Intervention Induced Mouse Model of Intestinal Neuronal Dysplasia
Source: Front Pediatr. 2022 Jan 14;9:795678. doi: 10.3389/fped.2021.795678 (PMC8796853; doi:10.3389/fped.2021.795678)

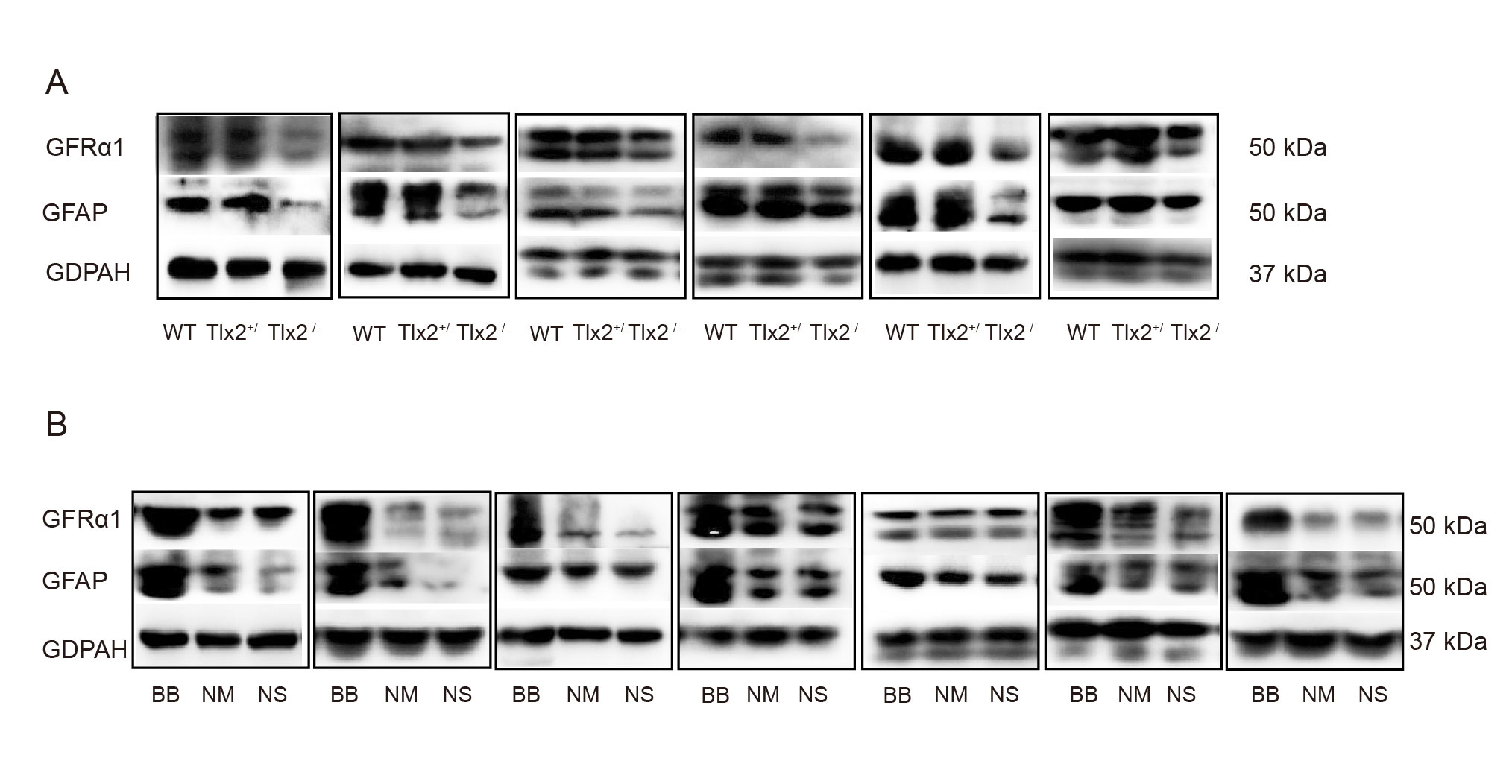

Supplement: Supplementary Figure 1 — (A) Western blot indicated that the protein expressions of GFAP and GFRα1 in colonic tissues of Tlx2−/− mice were decreased. (B) The protein expression of GFAP and GFRα in the colonic tissues of group BB was higher than those in the other two groups. [file Image_1.JPEG]
